# Effects of stimulus response compatibility on covert imitation of vowels

**DOI:** 10.3758/s13414-018-1501-3

**Published:** 2018-03-13

**Authors:** Patti Adank, Helen Nuttall, Harold Bekkering, Gwijde Maegherman

**Affiliations:** 1 Department of Speech, Hearing and Phonetic Sciences, University College London, Chandler House, 2 Wakefield Street, London, WC1N 1PF UK; 2 Department of Psychology, Lancaster University, Lancaster, LA1 4YF UK; 3 Donders Institute for Brain, Cognition and Behaviour, Radboud University, Nijmegen, the Netherlands

**Keywords:** Speech perception, Speech production, Multisensory processing

## Abstract

When we observe someone else speaking, we tend to automatically activate the corresponding speech motor patterns. When listening, we therefore covertly imitate the observed speech. Simulation theories of speech perception propose that covert imitation of speech motor patterns supports speech perception. Covert imitation of speech has been studied with interference paradigms, including the stimulus–response compatibility paradigm (SRC). The SRC paradigm measures covert imitation by comparing articulation of a prompt following exposure to a distracter. Responses tend to be faster for congruent than for incongruent distracters; thus, showing evidence of covert imitation. Simulation accounts propose a key role for covert imitation in speech perception. However, covert imitation has thus far only been demonstrated for a select class of speech sounds, namely consonants, and it is unclear whether covert imitation extends to vowels. We aimed to demonstrate that covert imitation effects as measured with the SRC paradigm extend to vowels, in two experiments. We examined whether covert imitation occurs for vowels in a consonant–vowel–consonant context in visual, audio, and audiovisual modalities. We presented the prompt at four time points to examine how covert imitation varied over the distracter’s duration. The results of both experiments clearly demonstrated covert imitation effects for vowels, thus supporting simulation theories of speech perception. Covert imitation was not affected by stimulus modality and was maximal for later time points.

**Electronic supplementary material:**

The online version of this article (10.3758/s13414-018-1501-3) contains supplementary material, which is available to authorized users.

Observing someone else perform an action has been shown to activate neural mechanisms required to perform that action (Buccino et al., 2004; Fadiga, Craighero, Buccino, & Rizzolatti, 2002). For speech, this type of *covert imitation* occurs whenever we hear and/or see someone speaking, and it involves activation of speech production mechanisms (Nuttall, Kennedy-Higgins, Devlin, & Adank, 2017; Nuttall, Kennedy-Higgins, Hogan, Devlin, & Adank, 2016; Watkins, Strafella, & Paus, 2003). Covert imitation processes are proposed to play a key role in current speech perception theories, commonly referred to as *simulation accounts* (Pickering & Garrod, 2013; Wilson & Knoblich, 2005). Simulation accounts propose that listening to speech results in automatic activation of the articulatory motor plans for producing speech. These motor plans consist of simulations of the movements of articulators that are generated while the listener is processing the incoming speech signal. The generated motor plans then inform forward models of the heard speech that run in parallel with the unfolding speech signal (Kawato, 1999). Forward models are thought to use implicit knowledge of the perceiver’s articulatory mechanics as a real-time mental simulation to track others’ speech that support speech perception. These mental simulations generate top-down predictions of incoming speech, serving as a prediction signal supporting perception and thereby streamlining interaction.

Covert imitation in speech can be demonstrated using neuroimaging methods including functional magnetic resonance imaging (fMRI), neurostimulation methods such as transcranial magnetic stimulation (TMS), or using behavioural paradigms. Using fMRI, it was demonstrated that passively listening to speech broadly activates speech production regions, including motor and premotor areas (Wilson, Saygin, Sereno, & Iacoboni, 2004). Areas in the primary motor cortex (M1) have been found to respond in a somatotopic manner during speech perception: Areas of M1 show activation congruent with the primary articulator producing the perceived speech stimulus. Pulvermüller et al. (2006) used fMRI to demonstrate that lip and tongue areas of M1 responded in a somatotopic manner when participants listened to sounds produced with the lips (/p/) and the tongue (/t/).

Using TMS, a causal link has been demonstrated between articulatory M1 and the efficacy of perception of sounds articulated using the congruent articulator (D’Ausilio et al., 2009; Möttönen & Watkins, 2009). D’Ausilio et al. (2009) administered TMS pulses to lip or tongue M1 while participants performed a discrimination task for sounds produced with the lips (/p/ and /b/) or tongue (/t/ and /d/) as active articulators. D’Ausilio et al. report a double dissociation in speech sound discrimination: Participants showed poorer discrimination for lips sounds, but not for tongue sounds, after a TMS pulse to the lips, and vice versa. Möttönen and Watkins (2009) asked participants to perform a categorical perception task of spoken syllables before administering 15 minutes of off-line repetitive TMS to lip M1. After receiving TMS, participants repeated the task and showed impaired categorical perception of syllables involving lip sounds (/pa/–/ba/ and /pa/–/ta/) but not tongue sounds (/ka/–/ga/, and /da/–/ga/).

Besides establishing causal links between a brain area and behaviour, TMS has also been used to estimate the relative excitability of the corticobulbar tract innervating speech muscles (Adank, Nuttall, & Kennedy-Higgins, 2016) while listening to speech. Following a TMS pulse to an area in articulatory M1, it is possible to record the resulting action potentials, motor evoked potentials (MEPs), in the corresponding muscle. Increased MEPs while perceiving speech can be regarded to imply covert imitation. This covert imitation response is also somatotopic in nature and, for instance, also reflects the clarity with which the speech stimulus was produced. Nuttall et al. (2016) measured MEPs from lip M1 while participants were listening to clearly spoken syllables (/apa/, /aba/, /ata/, and /ada/) and distorted syllables (produced with a tongue depressor in the speaker’s mouth). As in Möttönen and Watkins (2009) and D’Ausilio et al. (2009), participants showed somatotopic effects: Lip M1 was facilitated for lip sounds, and further facilitation was measured for distorted lip sounds. Moreover, Sato, Buccino, Gentilucci, and Cattaneo (2009) demonstrated that somatotopic effects extend to visual speech processing; they applied TMS to left tongue M1 and recorded MEPs from participants’ tongue muscles during perception of congruent and incongruent audiovisual syllables incorporating tongue-related and/or lip-related phonemes (visual and acoustic /ba/, /ga/, and /da/; visual /ba/ and acoustic /ga/; and visual /ga/ and acoustic /ba/). Greater excitability of tongue M1 was measured for syllables incorporating visual and/or acoustic tongue-related speech sounds, compared with the presentation of lip-related speech sounds.

Behaviourally, covert imitation can be measured using interference paradigms, such as the stimulus response compatibility (SRC) paradigm. SRC tasks were originally mostly used to study covert imitation of manual actions (Brass, Wohlsläger, Bekkering, & Prinz, 2000), but have also been used for speech stimuli. In a manual SRC task, participants are instructed to perform a manual action in response to a prompt (e.g., lift index finger when a written “1” appears, lift middle finger when “2” appears). The prompt is presented superimposed on a distracter: An image or video of a hand lifting the index or middle finger. When the prompt is presented in the presence of a congruent distracter (“1” with a video of a lifting index finger), participants are faster to perform the correct response than when the prompt is presented together with an incongruent distracter (“1” with a video of a lifting middle finger). For congruent distracters, it is assumed that action observation invokes motor patterns for performing the prompted action, thus reducing response times (RTs). In contrast, incongruent distracters result in competition between the activated motor patterns and those required to produce the prompted response, leading to slower RTs. A larger SRC effect (i.e., a larger RT difference between incongruent and congruent pairs) indicates that motor mechanisms were more activated for the distracter. SRC paradigms are thought to provide a fairly direct measure of the relative activation of motor mechanisms and of covert imitation (Heyes, 2011).

In speech SRC paradigms (Galantucci, Fowler, & Goldstein, 2009; Jarick & Jones, 2009; Kerzel & Bekkering, 2000; Roon & Gafos, 2015), the participant produces a speech response following a prompt (e.g., *ba*) while ignoring a distracter (e.g., a video of someone saying *da*). As reported for manual SRC studies, responses to the prompt are slower for incongruent (*da*) than for congruent (*ba*) distracters (Kerzel & Bekkering, 2000). Kerzel and Bekkering (2000) used video-only distracter stimuli, and later studies extended the use of the SRC paradigm to audio and audiovisual modalities. Jarick and Jones (2009) ran the SRC task with video-only, audio-only, and audiovisual distracters. Participants were required to respond by either pressing a button or speaking when seeing the prompt *ba* or *da*, in separate tasks. They measured the largest covert imitation effects for their video-only condition and the smallest effect for the audio-only condition for the speech response condition. They also report no covert imitation effects for manual responses (a pattern also reported in Galantucci et al., 2009), thus demonstrating that covert imitation is effector specific.

Converging evidence from fMRI, TMS, and behavioural studies thus indicates that observing visual, auditory, or audiovisual speech sounds results in covert imitation. However, covert imitation effects for speech sounds have only been demonstrated for a select class of speech sounds, that is, for stop consonants, either in a CV syllable or in isolation. It is not clear if observing vowels also invokes covert imitation, and if these effects would be comparable in size with covert imitation effects reported for consonants. A single fMRI study examined whether vowels are somatotopically represented in articulatory M1 (Grabski et al., 2013). Grabski et al. (2013) presented listeners with recordings of participants’ own monophthongal French vowels (/i y u e ø o ɛ œ ɔ/). These vowels varied in vowel height (close, midclose, and midopen), tongue position (front or back), and lip rounding (rounded or unrounded). If vowel articulation is represented somatotopically, as is the case for stop consonants, then it could be expected that tongue position and rounding could be linked to tongue and lip M1, respectively, and vowel height to the jaw muscle M1 representation. However, Grabski et al. report no activation in M1 related to vowel perception, and neural responses linked to vowel perception were diffusely distributed across a network of bilateral temporal, left prefrontal, and left parietal areas. Thus, to our knowledge, no fMRI, TMS, or behavioural SRC study has demonstrated that observers covertly imitate vowel stimuli.

There is evidence that consonants and vowel are processed differently at neural levels. Brain damage has been shown to impair consonant processing while preserving vowel processing and vice versa (Caramazza, Chialant, Capasso, & Miceli, 2000). Moreover, electrical stimulation of the temporal cortex in patients with aphasia impaired consonant discrimination, but not vowel discrimination (Boatman, Hall, Goldstein, Lesser, & Gordon, 1997; Boatman, Lesser, Hall, & Gordon, 1994). Results from fMRI studies also suggest a difference in the neural processing of consonant and vowel sounds (Seifritz et al., 2002). Using behavioural studies, further evidence was provided for a dissociation in the roles vowels and consonants play, in speech perception, specifically. Several perceptual phenomena occurring for stop consonants, such as categorical perception (Liberman, Harris, Hoffman, & Griffith, 1957) and duplex perception (Liberman, Isenberg, & Rakerd, 1981), were found to not extend to vowels (Gerrits & Schouten, 2004; Whalen & Liberman, 1996). Results from patient studies, electrical stimulation experiments, fMRI studies, and behavioural studies thus converge on the notion that consonants and vowels may be treated differently by the speech processing system. It is important to establish whether covert imitation occurs for stop consonants and for vowels, and if it does, whether there is a difference in the size of covert imitation effects. If covert imitation only occurs for (stop) consonants, and not for vowels, then this implies that listening to vowel sounds may not result in the automatic activation of articulatory motor plans required for generating simulations during speech perception.

The present study tested whether listeners covertly imitate vowels. Past studies used CV syllables where place of articulation or voicing was contrasted between the initial consonants, and the following vowel remained the same (Galantucci et al., 2009; Jarick & Jones, 2009; Kerzel & Bekkering, 2000; Roon & Gafos, 2015). In our CVC (consonant–vowel–consonant) stimuli, the consonants remained the same (/h/ and /d/), while the vowel was either /i/ (as in *heed*) or /ʊ/ (as in *hood*). The vowels in *heed* and *hood* were selected as they are produced with either spread (*heed*) or rounded lips (*hood*) and can thus be distinguished visually.

Using vowels also allows for more detailed scrutiny of variation over time in the covert imitation effect, as vowels are less transient than consonants. We therefore presented the prompt at four time points (stimulus onset asynchronies [SOAs]) during articulation. SOA manipulations were also used in Roon and Gafos (2015), Kerzel and Bekkering (2000), and Galantucci et al. (2009). However, all three studies used CV stimuli, and SOAs were restricted to a short time span (i.e., between 100 ms and 300 ms for Roon & Gafos [100 ms, 200 ms, 300 ms]; between zero and 500 ms for Kerzel & Bekkering [0 ms, 167 ms, 333 ms, 500 ms]; and between zero and 495 ms for Galantucci et al. [0 ms, 165 ms, 330 ms, 495 ms]). The SOAs used in past studies were spaced apart in equal intervals of the distracter video duration and not linked to specific articulatory features, such as the onset or offset of articulation. In the present study, we presented the prompts at four SOAs coinciding with the start of the distracter (0 ms, SOA1), the onset of visible articulation (335 ms, SOA2), the point where the auditory signal started and where the visual articulatory difference between the two vowels was maximal (670 ms, SOA3), and the point at which visible articulation ceased for both vowels (1,700 ms, SOA4). We expected smaller covert imitation effects for SOA1 compared with later SOAs, as no distracting articulatory information was present at zero ms. Previous studies found smaller or no interference effects when the SOA was set to the start of the trial. We included SOA2 and SOA4 to establish whether the covert imitation effect is larger at the beginning or the end of the articulatory sequence, and SOA3 to establish if the covert imitation effect is maximal when the visual difference between the two distracters is also maximal.

Finally, it is currently unclear how distracter modality affects covert imitation of vowels. A single previous study examined the effect of video, audio, and audiovisual distracter stimuli on covert imitation for consonants (Jarick & Jones, 2009). However, as Jarick and Jones (2009) presented the prompt at a single time point (100 ms from the start of the distracter stimulus), it remains unclear how modality affects covert imitation over time. The four SOAs will thus also serve to establish if and how distracter modality interacts with covert imitation over time.

## Experiment 1

### Method

An a priori power analysis (G*Power 3.1.9.2; Faul, Erdfelder, Lang, & Buchner, 2007) for a between-groups design with three groups and 240 observations per participant suggested a sample size of 66 participants (22 per group) with a Type I error of *p* < .05 and observed power of 80% for an expected effect size of 0.25. Sixty-six participants, 22 per group (46 females, 20 males, mean age = 22.4 years, *SD* = 4.8 years, range: 18–40 years) took part. One male participant from the audio group was excluded for not following task instructions. Participants were randomly assigned to three groups: video (16 females, six males, mean = 23.6 years, *SD* = 4.8 years, range: 18–40 years); audio (12 females, 11 males, mean = 23.1 years, *SD* = 3.7 years, range: 19–31 years); and audiovisual (18 females, four males, mean = 20.6 years, *SD* = 4.1 years, range: 18–28 years). All were native speakers of British English, who reported normal or corrected-to-normal vision, normal hearing, and no (history of) dyslexia. The study was approved by UCL’s Research Ethics Committee (#0599.001). Participants gave informed consent and received course credit or payment.

**Fig. 1:**
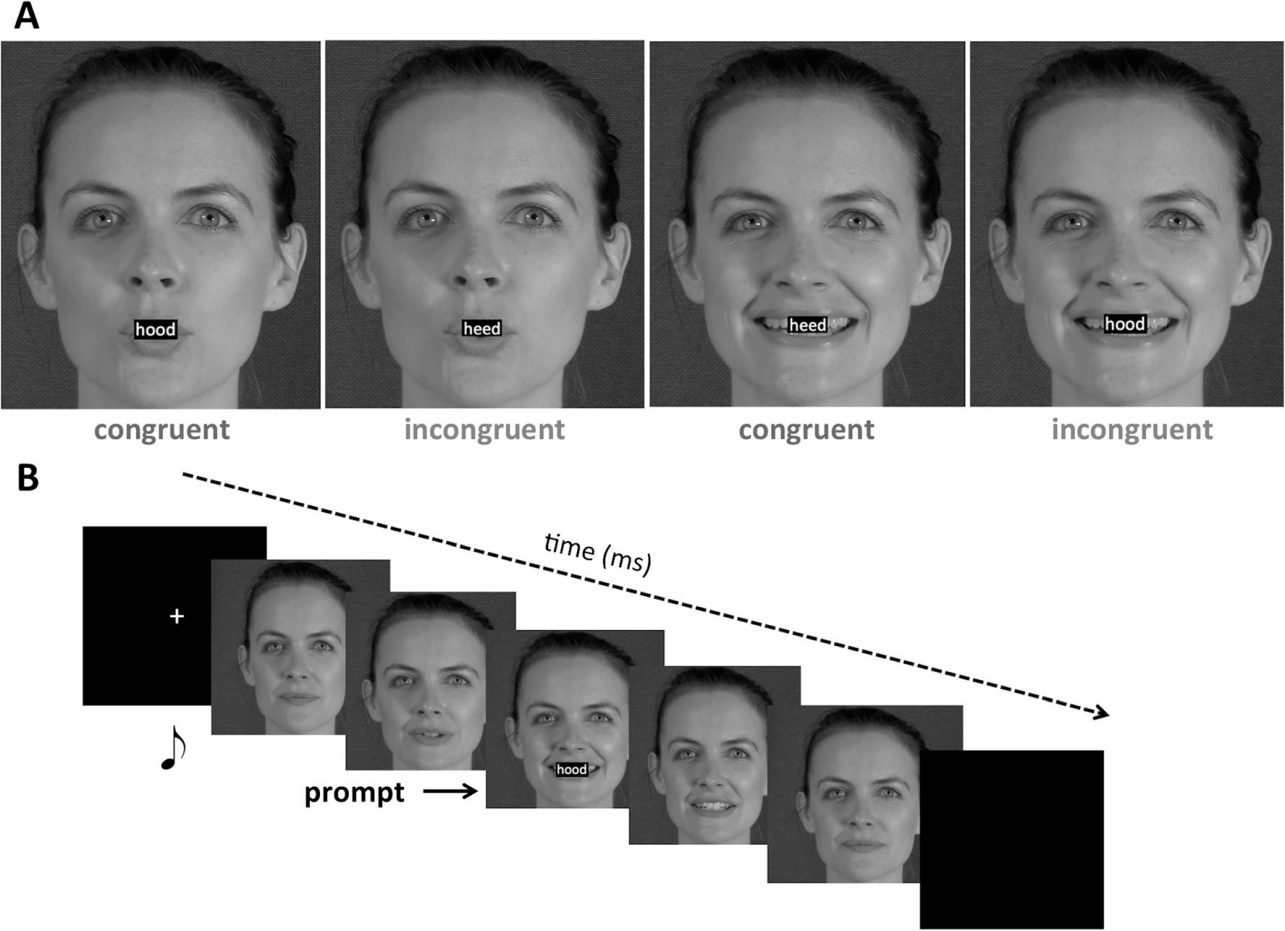
**a** Congruent trial for *hood* prompt, incongruent trial for *hood* prompt, congruent trial for *heed* prompt, incongruent trial for *heed* prompt. **b** Example of the timeline of an incongruent stimulus pair with *hood* prompt and *heed* distracter

The distracter stimuli consisted of two videos of a female speaker saying *heed* or *hood* (see Fig. 1). The video stimuli were recorded by a 29-year-old female speaker of British English, with a Canon Lagria HF G30 video camera on a tripod. The video recordings were edited using iMovie on an Apple iMac, and scaled down in resolution from 1920 × 1090 to 1280 × 720 in .avi format. The prompt was a jpeg image with a resolution of 300 dpi, 0.38 × 0.16 cm (45 × 19 pixels), was presented on-screen at a size of 1.1 × 0.5cm, and consisted of either *heed* or *hood* printed in boldfaced Arial font on a black background. Font size was adjusted so that the lip movements remained highly visible while the prompt appeared centred on the mouth (see Fig. 1). The audio stimuli were recorded simultaneously with the video recordings, using a RODE NO1-A Condenser Microphone, a Focusrite Scarlett 2i4 USB Computer Audio Interface preamplifier plugged into the sound card input of a Dell PC in a sound-attenuated room at 44.1 kHz with 16 bits. Audio recordings were amplitude normalized off-line, down-sampled to 22.050 kHz, and scaled to 70 dB SPL (sound pressure level) using Praat (Boersma & Weenink, 2003). The audio file for *hood* had a total duration of 977 ms (/h/ segment: 137ms; /o/: 732 ms; /d/: 108 ms), and the audio file for *heed* also had a total duration of 977 ms (/h/ segment: 133 ms; /ʊ/: 734 ms; /d/: 110 ms). The video files were muted using iMovie (9.0.9), and the video and audio files were combined in Presentation software when the trial was presented.

The experiment was conducted in a sound-attenuated and light-controlled booth. The stimuli appeared on a PC monitor located 70 cm away from the participant. Stimuli were presented using Presentation (Neurobehavioral Systems). Audio was played through Sennheiser HD25 SP-II headphones. Instructions were provided on-screen. Participants were instructed to look out for the prompt and speak the prompt aloud as fast as possible, ignoring the video in the background. Participants completed 16 familiarisation trials to ensure they performed the task as instructed and spoke at appropriate loudness levels while avoiding making any other sounds. The experimenter left the room after the familiarisation session.

Trials in the main experiment proceeded as follows. First, a black screen with a fixation cross was presented for either 500 ms, 750 ms, or 1,000 ms (jitter, following Kerzel & Bekkering, 2000). Next, a tone (500 Hz, 200 ms) was presented to signal the start of the trial. In the video condition, subsequently the video was presented with the sound muted. In the audio condition, a still image of the speaker with her mouth closed was presented in the background, and the sound file started 670 ms from the start of the trial. In the audiovisual condition, the video started playing at zero ms, and the sound file started playing 670 ms after the start of the video. Note that audible articulation of vowels in an /hVd/ context tends to follow visible articulation. The start time of the audio was selected as initial pilot testing revealed this time point to be optimal for a natural effect and this time point was placed approximately in between the points in time when the audio started for the original *heed* and *hood* audiovisual recordings.

In all conditions, the prompt appeared superimposed over the lips of the speaker for a duration of 200 ms (see Fig. 1). The prompt was presented at four SOAs, chosen to coincide with key points in the stimulus: zero ms (start of the trial), 335 ms (onset of visible articulation in the video and audiovisual conditions), 670 ms (the start of the auditory signal in all three conditions), and 1,700 ms (end of visible articulation). The video started and ended with the speaker’s lips closed, and no eye-blinks were present.

Responses were recorded via a voice key in Presentation, using a Rode microphone plugged into a Scarlett preamplifier connected to the PC’s USB input, from voice onset for 2,500 ms. Responses could be made from the start of the trial (i.e., the start of the video). RTs were measured from the onset of the prompt across for all three groups. When no response had been detected after 2,500 ms from the start of the video, participants received a *no response* warning. Stimulus lists were randomised for each individual participant, and the same randomised stimulus lists were used across successive participants in the three groups. The experiment lasted approximately 40 minutes. Data, stimulus materials, and program code can be found on the Open Science Network, under the name SRC_Vowels (https://osf.io/sn396/). We first converted the raw error percentages per participant to rationalized arcsine units, or RAUs (Studebaker, 1985), as this procedure is customary for proportional scales (e.g., Adank, Evans, Stuart-Smith, & Scott, 2009). Transforming the raw proportions to RAU ensures that the mean and variance of the data are relatively uncorrelated and that the data are on a linear and additive scale (Studebaker, 1985). After transforming the error percentages data to RAUs, we performed a three-factor repeated-measures ANOVA, with the transformed error rates as the dependent variable and with prompt (*heed* or *hood*), congruence (congruent or incongruent), SOA (SOA1–SOA4) as within-subjects factors, and listener group as a between-subjects factor for Experiment 1 and modality (video, audiovisual, audio) as an additional within-subjects factor for Experiment 2.

The factors congruence (congruent, incongruent), prompt (*heed*, *hood*), SOA (1–4), and modality (video, audio, audiovisual) were manipulated to explore changes in the response times in milliseconds (RT), and analysed in a repeated-measures ANOVA, controlled for nonsphericity (Huynh–Feldt), and post hoc tests were corrected for multiple comparisons (Bonferroni). RTs were log transformed before they were entered into the statistical analyses (Baayen, 2008). Only correct responses were analysed. Errors were responses that were too early (<200 ms) or late (>1,000 ms), following Jarick and Jones (2009), absent or partial responses, plus trials in which participants produced incorrect or multiple prompts. It was determined whether a participant had produced a correct or incorrect response by two phonetically trained listeners. Sound-file editing was conducted by a research assistant blind to the congruence condition.

### Results

Participants made 9.4% errors on average. Of the 15,600 responses in total, 1,460 were classed as errors and excluded: 228 (1.5%) were missed responses; 1,042 (6.7%) were too early or too late; and in 190 (1.2%) cases, participants produced the wrong prompt. The analysis of the errors showed main effects of prompt and SOA, and significant interactions for Prompt × Congruence, Prompt × SOA (see Table S1 in the Supplementary Materials). Analysis of the errors showed that participants made more errors for *heed* (10%) than *hood* (8%). Participants made more errors for SOA1 (19%) than for the other three SOAs (SOA2: 8%, SOA3: 7%, SOA4: 4%). Participants also made significantly more errors for congruent (12%) than incongruent (9%) pairs for *heed*, but not *hood* (8% congruent and 9% incongruent). Participants also made more errors for SOA1 for *heed* (22%) than for *hood* (16%). No congruence effects were found.

**Fig. 2:**
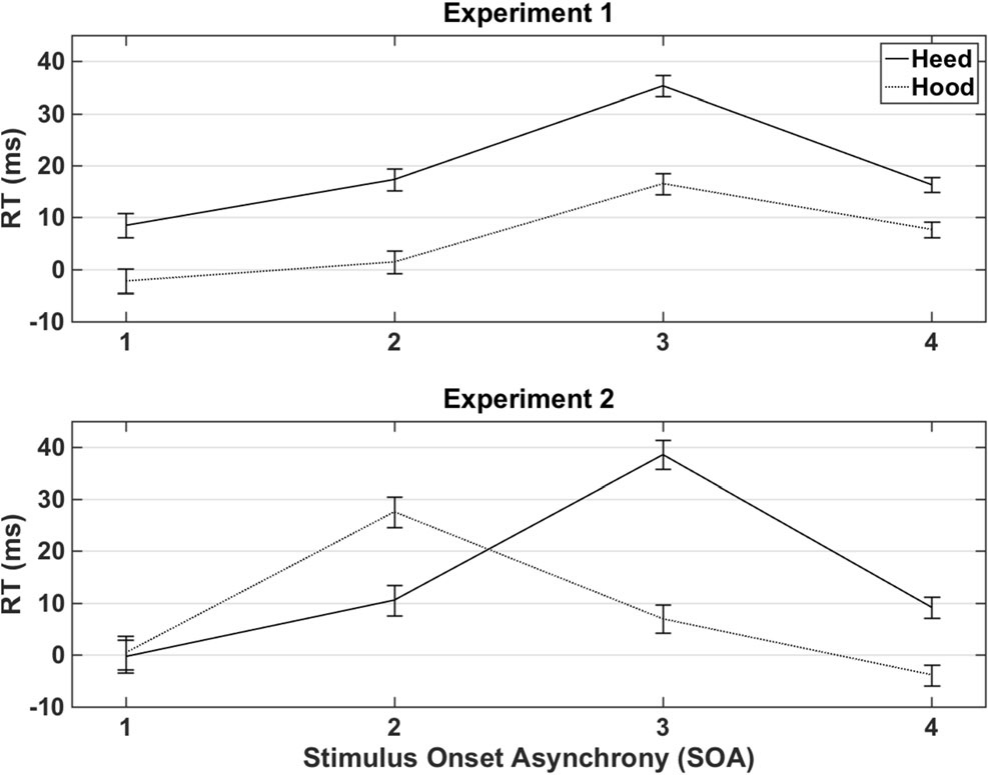
Difference scores in ms (incongruent minus congruent pairs) pooled across the video, audio, and audiovisual conditions, for each SOA and separated by prompt, for Experiment 1 (top) and Experiment 2 (bottom). Error bars represent one standard error

The analysis of the RTs included only correct responses. Main effects were found for prompt, congruence, SOA, and the following interactions: SOA × Modality, Prompt × Congruence, Prompt × SOA, and Congruence × SOA. Participants responded overall slower for *heed* than for *hood* prompts. The RTs showed an overall covert imitation effect, as RT were faster for congruent than incongruent trials (see Fig. 2, Table 1). As predicted, covert imitation effects differed per SOA and were largest for SOA3, and no covert imitation effect was found for SOA1. RTs were faster for later consecutive time points, except between SOA2 and SOA3. The SOA × Modality interaction was linked to slower responses for the video than for the audiovisual group, for SOA4 only. The Prompt × Congruence interaction was related to larger covert imitation effects for *heed* than for *hood. Heed* responses were slower than *hood* responses at SOAs 2 and 4. An analysis of difference scores (incongruent minus congruent RTs) showed that covert imitation effects were found for heed across all three groups, but for *hood* these effects were found for video and audio groups only.

**Table 1:** Averages plus standard deviations (in parentheses) for % error and response times in ms for congruent and incongruent stimulus pairs, per prompt, per stimulus onset asynchrony (SOA), and modality, for Experiment 1

			Errors			Response times		
			Video	Audio	Audiovisual	Video	Audio	Audiovisual
*Heed*	*Congruent*	SOA1	21 (41)	26 (44)	25 (44)	648 (123)	648 (123)	606 (140)
		SOA2	5 (23)	13 (33)	10 (30)	590 (115)	590 (115)	537 (130)
		SOA3	4 (20)	12 (33)	5 (22)	534 (103)	534 (103)	558 (144)
		SOA4	4 (19)	6 (24)	7 (26)	535 (85)	535 (85)	498 (109)
	*Incongruent*	SOA1	19 (39)	21 (41)	22 (42)	660 (131)	660 (131)	604 (136)
		SOA2	6 (23)	10 (30)	4 (20)	608 (106)	608 (106)	537 (145)
		SOA3	2 (15)	10 (29)	5 (23)	579 (111)	579 (111)	578 (130)
		SOA4	4 (19)	4 (19)	6 (24)	555 (75)	555 (75)	501 (100)
*Hood*	*Congruent*	SOA1	14 (34)	17 (38)	15 (36)	655 (134)	655 (134)	600 (119)
		SOA2	5 (21)	10 (30)	5 (23)	575 (110)	575 (110)	534 (129)
		SOA3	4 (19)	10 (31)	5 (23)	553 (107)	553 (107)	553 (139)
		SOA4	2 (15)	4 (20)	3 (17)	524 (72)	524 (72)	492 (139)
	*Incongruent*	SOA1	16 (37)	19 (39)	15 (36)	635 (125)	635 (125)	607 (130)
		SOA2	5 (23)	9 (29)	9 (28)	590 (119)	590 (119)	529 (138)
		SOA3	3 (16)	11 (31)	8 (28)	567 (102)	567 (102)	587 (149)
		SOA4	2 (13)	4 (19)	3 (18)	537 (80)	537 (80)	498 (104)

In conclusion, the results of Experiment 1 showed a clear main covert imitation effect for the response times only. Congruent trials were associated with faster responses than incongruent were trials across all three modalities. These results replicated earlier work showing effects of congruence for consonants in CV syllables (Jarick & Jones, 2009; Kerzel & Bekkering, 2000) and extended these effects to vowels in CVC syllables. However, the effects measured here were smaller than those for CV syllables (13 ms across all SOAs vs. ~35 ms for Experiment 1 in Kerzel & Bekkering, 2000, averaged across both prompts). Jarick and Jones (2009) report smaller covert imitation effects for audio than their video and audiovisual conditions. However, due to the between-group design, employed in Experiment 1, it was not feasible to directly establish the extent to which participants changed their responses under different modalities, as was done in Jarick and Jones (2009), who used a within-subjects design. Note that we chose to use a between-groups design in Experiment 1 to reduce the experimental duration (40 minutes) while optimising the number of trials per participant (240 per modality), and to avoid potential order effects from switching from one modality to the next. Experiment 2 used a within-groups design, in which all participants completed the task for all three modalities in separate blocks to further explore the effect of modality on covert imitation.

## Experiment 2

Experiment 2 aimed to independently replicate effects found in Experiment 1 using a within-groups design in which all participants completed the task for the three modalities in separate blocks.

### Method

An a priori power analysis for a within-groups design with 360 observations per participant suggested a sample size of 24 with a Type I error of *p* < .05 and observed power of 80%, for an expected effect size of 0.25. Twenty-four female participants (mean = 19.0 years, *SD* = 1.4 years, range: 18–23 years) took part in Experiment 2. None of these participants took part in Experiment 1. All participants were native speakers of British English, who reported normal or corrected-to-normal vision, normal hearing, and no (history of) dyslexia. Video data for one participant were missing due to a technical error. Materials, task, and general procedure were similar to Experiment 1, except that participants completed the three conditions video, audio, and audiovisual (120 trials each) in a counterbalanced order: Participant 1 first completed the video condition, followed by the audio and then the audiovisual conditions. The order for the next participant was audiovisual, video, audio, and the next participant completed the experiment in the order audio, audiovisual, video, in a single session lasting 60 minutes. The procedure was the same for all other participants. Stimulus lists were randomised per participant per condition, and the same randomised list was used across the three conditions per participant, per the procedure used in Experiment 1.

### Results

Participants made 8.5% errors overall. Of the 8,520 responses, 728 were classed as errors and excluded: 164 (1.9%) were missed responses, 417 (4.9%) were too early or too late, and in 147 (1.7%) cases participants produced the wrong prompt. Main effects were found for prompt, congruence, SOA, plus the Prompt × SOA interaction (see Table S2 in the Supplementary Materials). Participants made more errors for *heed* (10%) than for *hood* (7%). Participants made more errors for SOA1 (19%) than for the other SOAs (SOA2: 5%; SOA3: 5%; SOA4: 4%). Participants made fewer errors for congruent (8%) than for incongruent (9%) pairs. Participants also made more errors for SOA1 for *heed* (22%) than for *hood* (16%).

The analysis of the RTs included only correct responses. Main effects were found for congruence and SOA, plus the interactions Modality × SOA, Prompt × SOA, Congruence × SOA, and Prompt × Congruence × SOA interactions. An overall covert imitation effect was again found, as participants responded faster for congruent than for incongruent pairs. However, covert imitation effects were only found for SOA2 and SOA3, as the difference between incongruent and congruent trials was not significantly different for SOA1 and SOA4. Participants again responded overall faster for later consecutive SOAs. Modality × SOA interactions were rather inconsistent. Faster responses were recorded for audio SOA2 than for audiovisual SOA2; faster responses were found for video SOA3 than for audio SOA3; and faster responses were found for audio SOA4 than for video SOA4. Slower *heed* responses were reported for SOA1 and SOA2, but not for SOA3 and SOA4. No follow-up tests survived correction for the Prompt × Congruence × SOA interaction.

In conclusion, the results of Experiment 2 replicated the covert imitation effect for vowels reported for Experiment 1 for the response times and also reported a small covert imitation effect for the errors, which was not reported for Experiment 1. The results did not reveal an effect of distracter modality on covert imitation, even when participants performed the SRC task for all three modalities. Experiment 2 further showed a replication of the interaction between SOA and congruence; covert imitation was most prominent at SOA2 and SOA3 (Table 2).

**Table 2:** Averages plus standard deviations (in parentheses) for response times in ms for congruent and incongruent stimulus pairs, per prompt, per stimulus onset asynchrony (SOA), and modality, for Experiment 2

			Errors			Response times		
			Video	Audio	Audiovisual	Video	Audio	Audiovisual
*Heed*	*Congruent*	SOA1	20 (40)	24 (43)	20 (40)	20 (40)	671 (130)	670 (131)
		SOA2	4 (21)	6 (24)	7 (26)	4 (21)	582 (118)	608 (130)
		SOA3	3 (18)	5 (23)	9 (29)	3 (18)	537 (107)	561 (133)
		SOA4	6 (24)	2 (15)	5 (23)	6 (24)	529 (86)	513 (96)
	*Incongruent*	SOA1	28 (45)	25 (44)	28 (45)	28 (45)	661 (138)	685 (132)
		SOA2	5 (23)	6 (24)	4 (20)	5 (23)	618 (123)	632 (140)
		SOA3	2 (15)	7 (26)	8 (27)	2 (15)	575 (112)	593 (120)
		SOA4	5 (23)	6 (23)	4 (20)	5 (23)	529 (83)	524 (97)
*Hood*	*Congruent*	SOA1	14 (35)	11 (31)	15 (36)	14 (35)	639 (126)	652 (120)
		SOA2	5 (22)	4 (19)	7 (25)	5 (22)	590 (138)	604 (137)
		SOA3	2 (15)	2 (12)	7 (25)	2 (15)	569 (142)	581 (125)
		SOA4	3 (17)	4 (20)	4 (19)	3 (17)	499 (95)	519 (84)
	*Incongruent*	SOA1	16 (37)	17 (38)	16 (37)	16 (37)	635 (129)	659 (132)
		SOA2	2 (15)	4 (19)	10 (30)	2 (15)	573 (135)	612 (133)
		SOA3	2 (13)	7 (26)	9 (28)	2 (13)	589 (136)	586 (126)
		SOA4	2 (15)	2 (14)	6 (23)	2 (15)	507 (87)	511 (91)

## General discussion

This study aimed to establish whether observers covertly imitate vowel stimuli, how covert imitation varies over time, and how distracter modality affects covert imitation. We conducted two experiments in which participants produced vocal responses to a CVC prompt in the presence of a background distracter in video, audio, or audiovisual modalities. A clear covert imitation effect was found on the response times in both experiments; participants showed faster responses for congruent than for incongruent trials. Our study thus replicated earlier work that showed covert imitation effects on consonants (Galantucci et al., 2009; Jarick & Jones, 2009; Kerzel & Bekkering, 2000; Roon & Gafos, 2015) and extended these effects to vowels. We found covert imitation effects of 13 ms for Experiment 1 and 7 ms for Experiment 2, collapsed over the four SOAs. Kerzel and Bekkering (2000) report covert imitation effects of 35 ms for their Experiment 1, and Galantucci et al. (2009) report an effect of 28 ms for their Experiment 2. Covert imitation effects for vowels seem to be overall smaller than those reported for consonants. Observing incongruent vowel articulation may lead to less activation of articulatory motor patterns compared with observing incongruent stop consonant articulation. In the visual domain, the stop consonants generally used in SRC paradigms differ in the active articulator, namely, lips or tongue, while our vowel stimuli differed only in the use of the primary articulator (lips rounded or unrounded). A distracter employing a different effector could result in greater, more widespread activation of articulatory patterns than a distracter changing the use of a single effector. Alternatively, observing a congruent vowel distracter may not facilitate the production of the correct response as much as is the case for stop consonants, again due to differences in articulation between the two classes of speech sounds. Follow-up studies could address the issue of articulatory complexity, for instance, by exploring somatotopy of perceived vowel stimuli using TMS, specifically, by measuring MEPs from lip and tongue muscles. Previous work has demonstrated somatotopy in tongue M1 (Sato et al., 2009) and lip M1 (Nuttall et al., 2017; Nuttall et al., 2016) congruent with the primary articulator of the observed speech sound. Somatotopy in TMS speech perception studies refers to the notion that specific parts of articulatory M1 become active, or show relative facilitation, when listening to speech sounds articulated using a congruent articulator (so lip M1 becomes relatively facilitated for lip-produced sounds such as /t/ or /d/). By comparing relative facilitation of lip M1 and tongue M1 while observing lip-articulated (/p/), tongue-articulated (/t/) sounds with unrounded (/i:/), and rounded vowels (/ʊ/ or /y/ for languages other than British English, e.g., Dutch), it could be established if greater differences in facilitation occur for lip or tongue sounds.

Modality did not directly affect covert imitation, as no evidence was found of an interaction between congruence, modality, and SOA in either experiment. It must be concluded that modality effects on covert imitation seem to be moderate or small for vowels, replicating and extending past findings by Jarick and Jones (2009) for consonants.

Covert imitation effects were largest for SOA3 (26 ms) in Experiment 1, and SOA2 (20 ms) and SOA3 (23 ms) in Experiment 2. These results illustrate that covert imitation is maximal for the time point (670 ms) at which the difference between the two distracters is maximal visually (in the video and audiovisual conditions) and/or when the audio starts playing (in audio and audiovisual conditions). The absence of a covert imitation effect at SOA1 (0 ms) in either experiment shows that distracting audio and/or visual distracter information was required to elicit covert imitation effects. Participants also responded faster for later onsets in both experiments; a result also reported by Kerzel and Bekkering (2000) and Galantucci et al. (2009). Interference effects also differed across SOAs. For Experiment 1, interference effects were largest for SOA3 (26 ms), while for Experiment 2 these were largest for SOA2 (22 ms) and SOA3 (14 ms), and no interference effect was found at SOA1 in either experiment. Note that SOA3 (670 ms) was chosen to coincide with the moment at which the audio signal started in the audio and audiovisual modalities and also the point at which the visual difference between the two distracters was maximal (spread vs. rounded lips).

Covert imitation effects differed depending on the stimulus prompt; larger effects were found for *heed* than *hood,* in analogy with Kerzel and Bekkering (2000), who report a trend towards smaller effects for /ba/ than for /da/ prompts. Larger interference effects for *heed* imply more interference from *hood* and vice versa. Larger effects for *heed* (with a *hood* distracter) showed that a distracter with rounded lips results in more covert imitation than the other way around. Alternatively, lip rounding might be more visually salient than lip spreading, and as a result might subsequently lead to more activation of motor substrates. Alternatively, it seems possible that the conflict between prompt and distracter resulted in a perceived fusion between the distracter and prompt. Results from previous work has shown that observing conflicting audiovisual information can lead to perceived vowel fusions (Traunmüller & Öhrström, 2007). Traunmüller and Öhrström (2007) found that acoustic /geg/ dubbed onto visually presented /gyg/ was predominantly perceived as /gøg/. In Traunmüller and Öhrström’s study, visual lip rounding affected the auditory perception of spreading more than the degree to which visual perception of lip spreading affected the auditory perception of lip rounding. It seems possible that similar asymmetric partial fusions occur for conflicts between speech production and simultaneously presented distracters and that such asymmetric partial fusions can explain the difference in how participants perceived our incongruent prompt–distracter pairings. Finally, participants could have found the video that involved lip spreading (heed) more visually salient than the lip-rounding video (*hood*). Potential effects of the relative salience of lip spreading versus lip rounding warrants further investigation in future studies.

For both experiments, on average, 9% errors were found. Participants made more errors for *heed* than for *hood* prompts in both experiments. Error percentages were higher than those reported in previous work (Galantucci et al., 2009; Jarick & Jones, 2009; Kerzel & Bekkering, 2000; ~1%–3% across all three studies). Close inspection of the results showed that, for both experiments, most errors were due to participants failing to respond, or failing to respond on time, for SOA1 (0 ms), possibly as a result of missing the prompt altogether for this SOA. Jarick and Jones (2009) did not include trials in which the prompt was presented at the very start of the trial; the prompt was presented around 100 ms into the trial duration, so participants were more likely to not miss the prompt. Kerzel and Bekkering (2000) and Galantucci et al. (2009) showed the prompt at zero ms, but do not provide detailed information on how errors were distributed across SOAs. Finally, it is unclear whether error percentages in previous work included incorrect responses (i.e., the wrong prompt) or whether they only included early or late or missed responses (e.g., Experiments 2 and 3 in Galantucci et al.).

In conclusion, our study provides the first experimental evidence of covert imitation for vowels. Covert imitation effects for vowels were smaller than those previously reported for stop consonants, which may be due to less activation of articulatory motor plans during perception of vowel stimuli. Future studies could explore the possibility raised by our results that the dampened covert imitation effects for vowels compared to previously reported effects for consonants could be due to greater similarity between vowel stimuli than between contrastive stop consonants. Covert imitation of vowels is not modulated by stimulus modality, and appears linked to differences between distracter and prompt. We replicated this finding in two experiments. Our study thus supports simulation theories of speech perception, by clearly showing that perceiving vowels links to activation of speech motor mechanisms. Current theories (Pickering & Garrod, 2013; Wilson & Knoblich, 2005) predict that observing an action activates articulatory plans congruent with the observed action in a somatotopic fashion, based on the results of studies mostly using stop consonants. Past work has so far not demonstrated that vowel stimuli are processed in a similar somatotopic manner (Grabski et al., 2013). The lack of evidence of somatotopic processing for vowels in combination with our reported smaller covert activation effects imply that the type of articulatory plan activated during perception differs for different classes of speech sounds.

## Electronic supplementary material

ESM 1 (DOCX 16.6 kb)
