# Supplementary material for: Effects of stimulus response compatibility on covert imitation of vowels
Source: Atten Percept Psychophys. 2018 Mar 13;80(5):1290–9. doi: 10.3758/s13414-018-1501-3 (PMC6060983; doi:10.3758/s13414-018-1501-3)
Supplement: Supplementary file 1 — (DOCX 16.6 kb) [file 13414_2018_1501_MOESM1_ESM.docx]

Supplementary materials

Table B. Results of the repeated measures ANOVAs on the errors transformed to Rationalised Arcsine Units (RAU) and log-transformed (LogRT) Response Times from Experiment 1. Significant results are indicated with ‘*’.

|  | RAU | | | | LogRT | | | |
| --- | --- | --- | --- | --- | --- | --- | --- | --- |
| Factor | df | F | *p* | *η_2pa_* | df | F | *p* | *η_2par_* |
| *Prompt* | **1, 62** | **7.94** | **0.006*** | **0.11** | **1, 62** | **8.67** | **0.005*** | **0.12** |
| *Prompt×Modality* | 2, 62 | 0.09 | 0.917 | 0 | 2, 62 | 0.34 | 0.712 | 0.01 |
| *Congruence* | 1, 62 | 0.07 | 0.796 | 0 | **1, 62** | **42.45** | **<0.001*** | **0.41** |
| *Congruence×Modality* | 2, 62 | 1.48 | 0.236 | 0.05 | 2, 62 | 2.16 | 0.12 | 0.07 |
| *SOA* | **2.56, 159.57** | **93.75** | **<0.001*** | **0.60** | **2.84, 176** | **250.61** | **<0.001*** | **0.80** |
| *SOA×Modality* | 6, 186 | 1.43 | 0.206 | 0.04 | **6, 186** | **14.28** | **<0.001*** | 0.32 |
| *Prompt×Congruence* | **1, 62** | **4.39** | **0.04*** | **0.07** | **1, 62** | **11.76** | **0.001*** | 0.16 |
| *Prompt×Congruence× Modality* | 2, 62 | 0.63 | 0.536 | 0.02 | 2, 62 | 4.57 | 0.01* | 0.13 |
| *Prompt×SOA* | **3, 185.73** | **6.27** | **0.001*** | **0.09** | **3, 186** | **4.01** | **0.01*** | **0.06** |
| *Prompt×SOA×Modality* | 6, 186 | 1.50 | 0.184 | 0.05 | 6, 186 | 0.54 | 0.77 | 0.02 |
| *Congruence×SOA* | 3, 186 | 0.60 | 0.615 | 0 | **3, 186** | **9.5** | **<0.001*** | **0.13** |
| *Congruence×SOA× Modality* | 6, 186 | 0.99 | 0.434 | 0.03 | 6, 186 | 1.44 | 0.20 | 0.04 |
| *Prompt×Congruence×SOA* | 3, 184.98 | 0.27 | 0.844 | 0 | 3, 186 | 0.78 | 0.50 | 0.01 |
| *Prompt×Congruence× SOA×Modality* | 6, 184.98 | 0.28 | 0.945 | 0.01 | 6, 86 | 0.86 | 0.53 | 0.03 |

Table B. Results of the repeated measures ANOVAs on the errors transformed to Rationalised Arcsine Units (RAU) and log-transformed (LogRT) Response Times from Experiment 2. Significant results are indicated with ‘*’.

|  | RAU | | | | LogRT | | | |
| --- | --- | --- | --- | --- | --- | --- | --- | --- |
| *Factor* | df | F | *p* | *η_2pa_* | df | F | *p* | *η_2par_* |
| *Modality* | 2, 44 | 1.33 | 0.287 | 0.06 | 2, 44 | 2.93 | 0.06 | 0.12 |
| *Prompt* | **1, 22** | **8.38** | **0.008*** | **0.28** | 1, 22 | 1.31 | 0.26 | 0.06 |
| *Congruence* | **1, 22** | **5.80** | **0.025*** | **0.21** | **1, 22** | **23.41** | **<0.001*** | **0.52** |
| *SOA* | **3, 66** | **34.22** | **<0.001*** | **0.61** | **2.54, 55.8** | **130.19** | **<0.001*** | **0.86** |
| *Modality×Prompt* | 2, 44 | .623 | 0.541 | 0.03 | 2, 44 | 0.58 | 0.56 | 0.03 |
| *Modality×Congruence* | 2, 44 | 1.293 | 0.541 | 0.06 | 2, 44 | 1.07 | 0.35 | 0.05 |
| *Prompt×Congruence* | 1, 22 | 0.03 | 0.955 | 0 | 1, 22 | 1.51 | 0.23 | 0.06 |
| *Modality×Prompt× Congruence* | 2, 44 | 0.85 | 0.435 | 0.04 | 2, 44 | 2.78 | 0.07 | 0.11 |
| *Modality×SOA* | 4.62, 101.59 | .215 | 0.808 | 0.01 | **6, 132** | **12.91** | **<0.001*** | **0.37** |
| *Prompt×SOA* | **3, 66** | **5.795** | **0.001** | **0.21** | **2.54, 5.91** | **5.5** | **0.004*** | **0.2** |
| *Modality×Prompt× SOA* | 6, 132 | 0.912 | 0.488 | 0.04 | 6, 132 | 0.8 | 0.57 | 0.04 |
| *Congruence×SOA* | 3, 66 | 1.519 | 0.218 | 0.02 | **3, 66** | **4.95** | **0.004*** | **0.18** |
| *Modality×Congruence× SOA* | 4.38, 96.296 | 0.51 | 0.745 | 0.02 | 6, 132 | 1.88 | 0.09 | 0.08 |
| *Prompt×Congruence× SOA* | 3, 66 | 1.207 | 0.314 | 0.05 | **3, 66** | **4.31** | **0.008*** | **0.16** |
| *Modality×Prompt× Congruence×SOA* | 6, 132 | 0.767 | 0.597 | 0.03 | 6, 132 | 0.85 | 0.53 | 0.04 |
